# Hypothetical applications of MR-guided focused ultrasound in central nervous system tuberculosis: blood–brain barrier disruption, granulomatous lesion modulation, targeted drug delivery, and immune microenvironment reprogramming

**DOI:** 10.1097/MS9.0000000000005162

**Published:** 2026-08-21

**Authors:** Shreya Singh Beniwal, Chimuka Mwaanga, Mohammed Jassim, Akash Rawat, Prashasti Dahiya, Rafael Everton Assunção Ribeiro da Costa, Anju Pandey, Anam Sayed Mushir Ali, Jonathan Moncada, Shaikh Sumaiya Abdul Mateen, Aarushi Mishra

**Affiliations:** a Lady Hardinge Medical College, New Delhi, India; b National Forensic Authority, Lusaka, Zambia; c Thirumala Devaswom Medical College (TDMC), Alappuzha, Kerala, India; d Himalayan Institute of Medical Sciences, Swami Rama Himalayan University, Dehradun, Uttarakhand, India; e ESIC Medical College and Hospital, Faridabad, India; f Universidade Estadual de Campinas (UNICAMP), Campus Cidade Universitária Zeferino Vaz, Campinas, São Paulo, Brazil; g Memorial Sloan Kettering Cancer Center, New York, NY, United States; h Indian Institute of Medical Science and Research, Jalna, India; i St. Francis Medical Center, Los Angeles, California, United States; j AMA School of Medicine, Makati City, Philippines; k Danylo Halytsky Lviv National Medical University, Lviv, Ukraine

**Keywords:** blood–brain barrier disruption, CNS tuberculosis, granulomatous lesion modulation, MR-guided focused ultrasound, precision neurotherapeutics

## Abstract

Central nervous system tuberculosis (CNS-TB) comprises tuberculous meningitis (TBM), parenchymal tuberculomas, and spinal arachnoiditis. It accounts for 5% to 10% of extrapulmonary TB and is more common in immunocompromised individuals, particularly those with HIV. Despite early diagnosis and treatment, mortality remains high, exceeding 30% in TB meningitis, and, as a consequence, many survivors develop cognitive, motor, or visual deficits. Management has proven to be difficult due to the blood–brain barrier and blood–cerebrospinal fluid barrier, which limit drug penetration, especially in early or non-inflammatory stages. Even after disruption of these barriers owing to inflammation, drug delivery remains inconsistent and often fails to reach the required concentrations. Tuberculomas further compound this problem due to their granulomatous, avascular, and fibrotic architecture, which protects *Mycobacterium tuberculosis* from both host immunity and antibiotics. Magnetic resonance–guided focused ultrasound (MRgFUS) is an established, non-invasive neurosurgical technology that may offer a platform to transiently disrupt physiological barriers or modulate lesion structure using microbubble-mediated cavitation. Although MRgFUS is FDA-approved for movement disorders and under investigation in brain tumors, its application in CNS-TB remains entirely untested. Conceptually, MRgFUS could enhance drug delivery to protected CNS compartments, theoretically alter granulomatous architecture to improve antimicrobial penetration, and facilitate targeted delivery of immunomodulatory agents. This narrative and hypothesis-generating review explores the theoretical role of MRgFUS as an adjunctive strategy in CNS-TB, while explicitly acknowledging current evidence gaps and safety uncertainties.

## Introduction

Central nervous system tuberculosis (CNS-TB) is a severe form of extrapulmonary tuberculosis, encompassing tuberculous meningitis (TBM), intracranial tuberculomas, and spinal arachnoiditis^[^1,2^]^. Although CNS involvement represents only 5–10% of extrapulmonary TB cases, it is associated with disproportionately high morbidity and mortality, particularly among immunocompromised individuals, including those living with HIV^[^3^]^. TBM remains the most lethal presentation, with reported mortality rates exceeding 30% and a substantial proportion of survivors suffering from long-term neurological impairments that significantly reduce functional independence and quality of life^[^3^]^. These outcomes highlight the limitations of current therapeutic approaches and the need for innovative adjunctive strategies.

HIGHLIGHTS

- Magnetic resonance–guided focused ultrasound (MRgFUS) enables non-invasive, real-time modulation of central nervous system (CNS) tuberculous lesions under MRI guidance.
- Transient opening of the blood–brain and blood–CSF barriers by MRgFUS enhances targeted anti-tubercular drug delivery.
- Granulomatous and fibrotic CNS lesions can be mechanically disrupted, thereby improving antimicrobial and immune cell penetration.
- MRgFUS may reprogram the CNS immune microenvironment, reducing neuroinflammation and long-term neurological deficits.
- Integration of MRgFUS with conventional therapy offers a precision-targeted approach for improved CNS-TB management.

One of the major obstacles in CNS-TB management is inadequate penetration of anti-tubercular drugs across the blood–brain barrier (BBB) and the blood–cerebrospinal fluid barrier (BCSFB)^[^4^]^. During early or minimally inflammatory stages of disease, these barriers often remain intact, resulting in insufficient drug concentrations within infected neural tissues. This pharmacological sequestration is particularly relevant in granulomatous lesions such as tuberculomas, which respond poorly to systemic therapy^[^5^]^. Beyond direct mycobacterial injury, host immune responses play a critical role in neurological damage, with dysregulated inflammation contributing to vasculitis, infarction, and long-term neurocognitive impairment^[^6^]^. Therefore, therapeutic strategies in CNS-TB must balance antimicrobial efficacy with control of immune-mediated injury.

Magnetic resonance–guided focused ultrasound (MRgFUS) has emerged as a promising non-invasive neuromodulatory technology with potential therapeutic applications in neurological and oncological disorders.

MRgFUS enables precise MRI-guided targeting of intracranial regions and can induce controlled thermal or mechanical effects^[^7^]^. Low-intensity focused ultrasound (LIFU), when combined with microbubbles, has been shown to transiently and reversibly open the BBB in both preclinical and clinical studies^[^8,9^]^.

**Figure 1.**
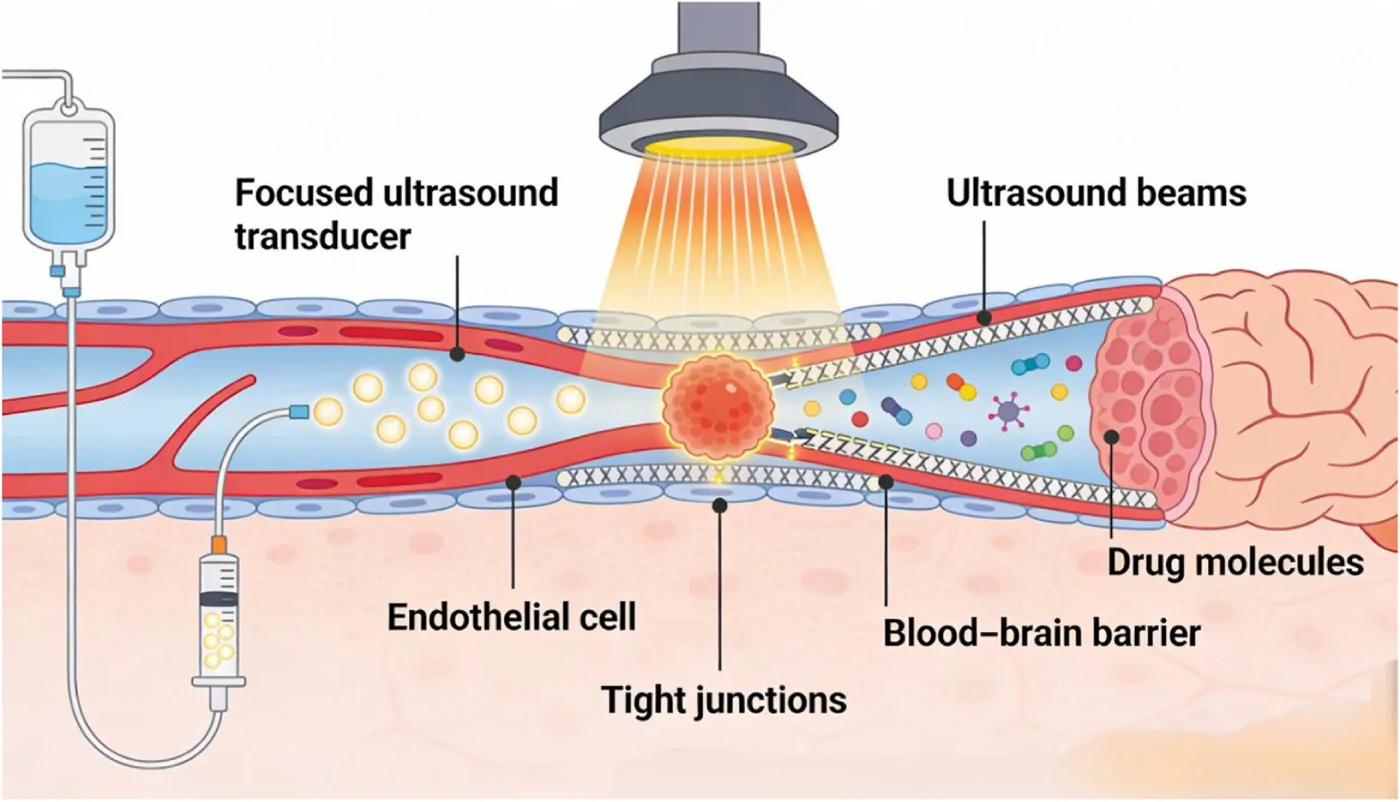
Focused ultrasound–mediated modulation of intracranial lesions.

As shown in Figure 1, MRgFUS encompasses both high-intensity focused ultrasound (HIFU), which produces localized thermal ablation^[^10–12^]^, and LIFU, which facilitates transient BBB disruption for targeted drug delivery^[^13–15^]^. This review evaluates MRgFUS as an adjunctive platform for CNS-TB, focusing on its potential to enhance anti-tubercular drug penetration, modify granulomatous lesions, and enable targeted host-directed or immunomodulatory therapies^[^16^]^.

Although MRgFUS has demonstrated safety and feasibility in neurological and oncological conditions, no experimental or clinical studies have evaluated its use in CNS-TB. Consequently, all proposed applications discussed herein should be regarded as conceptual extrapolations rather than evidence-based interventions.

Schematic illustration of focused ultrasound–based therapy demonstrating systemic administration of inert, gas-filled microbubbles followed by targeted ultrasound application. Acoustic activation of the microbubbles induces controlled cavitation, resulting in transient disruption of endothelial tight junctions and reversible opening of the BBB, thereby facilitating localized drug delivery to pathological tissue. Adapted from Rathi *et al*^[^17^]^.

## Literature search strategy and scope

This manuscript is a narrative, hypothesis-generating review. The literature was identified through non-systematic searches of PubMed, Scopus, and Google Scholar, focusing on publications from approximately 2000 through 2025. Search terms included combinations of “central nervous system tuberculosis,” “tuberculoma,” “blood–brain barrier,” “focused ultrasound,” “MRgFUS,” “drug delivery,” and “immunomodulation.” Studies addressing MRgFUS in oncology, neurodegeneration, brain abscesses, and other CNS disorders were included when mechanistically relevant. No formal PRISMA workflow or meta-analytic synthesis was performed, consistent with the conceptual scope of this review.

## Strengths of the present review

This narrative review provides a conceptual framework for exploring the potential role of MR-guided focused ultrasound in the management of CNS-TB, a field in which no prior focused synthesis exists. First, the review integrates insights from neuro-oncology, neuromodulation, and infectious disease research to propose a translational application of MR-guided focused ultrasound for infectious granulomatous pathology. Second, it highlights pharmacokinetic limitations of current antitubercular therapy in the CNS and discusses MRgFUS-mediated BBB modulation as a potential strategy to enhance drug delivery. Third, the review introduces possible mechanisms through which focused ultrasound may influence granulomatous lesions and inflammatory microenvironments. Finally, the manuscript synthesizes multidisciplinary evidence to stimulate future experimental and clinical investigations.

## Pathological aspects of CNS-TB

CNS-TB leads to a range of pathological conditions, encompassing basal leptomeningitis, vasculitis and stroke, hydrocephalus, cranial nerve involvement, myeloradiculopathy, hypothalamic syndrome, brain abscesses, and tuberculomas^[^18^]^. Hydrocephalus was observed in nearly 40% of patients, and stroke was reported in 30–50% of patients with TBM; both complications were associated with poor functional outcomes and mortality^[^18,19^]^. Tuberculomas and cerebral vasculitis also cause mass effect, focal neurological deficits, seizures, and long-term disability^[^18,20^]^. Other non-neurological complications include hyponatremia and diabetes insipidus^[^18^]^. In summary, tuberculosis of the brain is lethal if undiagnosed and untreated, leads to severe disability, and poses a health burden to society^[^21^]^, necessitating the development of novel image-guided focal interventions such as MRgFUS.

## MRgFUS for granulomatous lesion modulation in CNS-TB

CNS tuberculoma is one of the three forms of CNS-TB, along with TBM and spinal arachnoiditis. Hematogenous dissemination of the primary TB infection leads to deep-seated granulomatous foci in the CNS, which may coalesce into tuberculomas. These may be detected incidentally on imaging as single or multiple lesions, or present with symptoms of space-occupying lesions such as headache, papilledema, and neurological deficits^[^22^]^. Granulomas may be caseating or noncaseating^[^23^]^. Histologically, they are encapsulated epithelioid granulomas composed of macrophages, Langhans giant cells, lymphocytes, plasma cells, and fibroblasts arranged in organized layers^[^22^]^. Tuberculomas are avascular masses with surrounding edema^[^24^]^. Their poor vascularization limits drug delivery into their necrotic core, allowing bacteria to persist^[^25,26^]^. They can occur anywhere in the brain, including the supratentorial region, brainstem, posterior fossa, sella, or spinal cord^[^27^]^.

**Figure 2.**
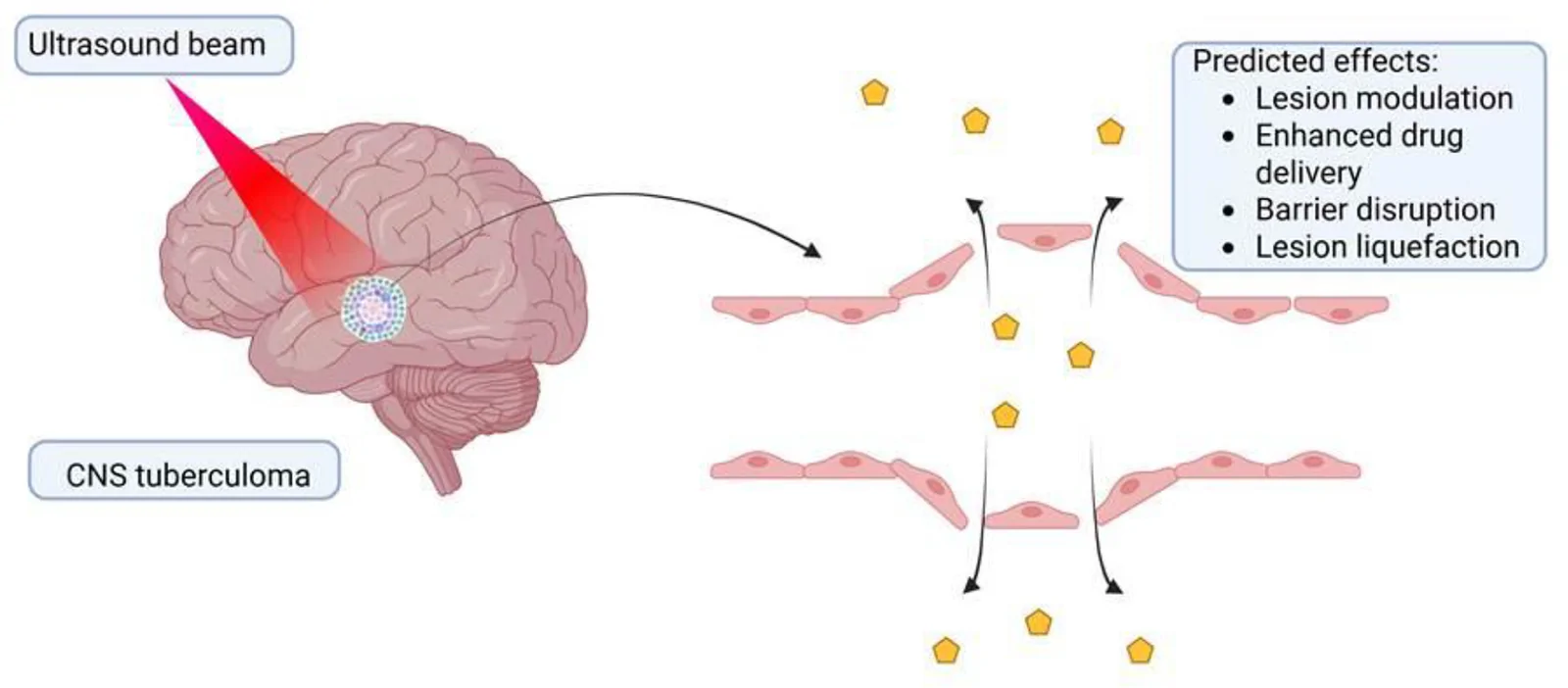
Conceptual schematic illustrating hypothetical MRgFUS effects on a CNS tuberculoma.

MRgFUS, a form of HIFU, has been successfully applied in several intracranial disorders, including epilepsy, ventriculostomy, septum pellucidotomy, trigeminal neuralgia, and targeted CNS drug delivery^[^28^]^ with notable use in Parkinsonism treatment^[^29^]^. No published study has specifically applied MRgFUS/HIFU to CNS tuberculomas. Similarly, a 2021 review highlighted the absence of focused ultrasound for TB or HIV drug delivery in the brain^[^30^]^. Preclinical models provide indirect support for ultrasound-enhanced approaches^[^31–33^]^. Rieck *et al* showed that MRgFUS (3 Hz) reduced bacterial counts in a mouse model of MRSA subcutaneous abscesses^[^32^]^; Liu *et al* demonstrated that low-intensity FUS with microbubbles could open the BBB, modulate immunity, contain infection, and reduce neuronal injury in a rodent brain abscess model^[^33^]^; and Xie *et al* reported on HIFU against Bacillus Calmette–Guérin *in vitro* and *in vivo*^[^31^]^. These studies suggest potential relevance to TB, although no experiments have targeted CNS tuberculomas directly (Fig. 2).

MRgFUS, particularly in its HIFU form, has been successfully applied to dense, fibrotic, and avascular tissues in non-infectious conditions, including uterine fibroids and intracranial tumors^[^10–12^]^. However, no studies have evaluated MRgFUS or HIFU in CNS tuberculomas.

Hydrocephalus is a frequent complication of TBM, caused by granulomatous inflammatory exudate obstructing cerebrospinal fluid (CSF) flow^[^34^]^. No clinical trials have used MRgFUS for hydrocephalus. An experimental preclinical study performed third ventriculostomy using MRgFUS in pigs and in cadaveric models, suggesting feasibility for septum pellucidotomy. While untested in TBM-associated hydrocephalus, the ability of MRgFUS to create non-invasive CSF pathways warrants exploratory evaluation^[^35^]^. Another cadaveric study used MRgFUS to create a precise lesion in the septum pellucidum or the wall of an enlarging CSF pocket to relieve hydrocephalus. The investigators demonstrated that the rapid temperature drop-off and high accuracy of MR-guided lesioning make MRgFUS a promising tool for establishing communication between CSF spaces^[^28^]^. Although no studies have applied MRgFUS to hydrocephalus secondary to TBM, its capacity to create non-invasive CSF pathways suggests that this approach could be theoretically explored in such settings.

However, this technique also carries important limitations and risks. The physical properties of the skull restrict the ability to accurately direct ultrasound beams toward the convexity, making centrally located intracranial targets more feasible for MRgFUS. Additionally, complications such as hemorrhage and the potential for imprecise targeting – with resulting thermal effects extending beyond the intended focal region – remain significant concerns that require rigorous evaluation^[^36^]^.

This original schematic, created by the authors, illustrates theoretical mechanisms by which MRgFUS might influence granulomatous lesions, including barrier modulation and enhanced drug penetration. No experimental data currently support these effects in CNS-TB.

## Blood–CSF/BBB disruption and enhanced drug delivery

CNS-TB typically presents in one of three clinicopathological forms: TBM, CNS tuberculoma, and spinal arachnoiditis. The cornerstone of therapy remains standard antituberculous treatment (ATT), most commonly a four-drug regimen consisting of isoniazid, rifampin, pyrazinamide, and ethambutol, often co-administered with corticosteroids or other anti-inflammatory agents^[^22^]^. However, effective management is challenged not only by emerging drug resistance but also by the limited ability of many anti-tubercular drugs to traverse the brain’s two major protective vascular interfaces – the BCSFB and the BBB^[^22,37^]^.

Among first-line agents, isoniazid and pyrazinamide exhibit excellent CSF penetration, achieving CSF levels comparable to those in plasma. In contrast, rifampin and ethambutol penetrate the BBB poorly, with rifampin CSF levels at only 10–20% of plasma concentrations. The current World Health Organisation (WHO) guidelines for the treatment of TBM are largely based on the guidelines for treating pulmonary TB and thus do not effectively account for the poor penetration of these drugs into the CSF^[^34^]^. This challenge is even greater in multi-drug resistant CNS-TB (MDR CNS-TB), where data on optimal drug regimens are limited. Some second-line drugs do have good CNS penetration, including fluoroquinolones, ethionamide, cycloserine, linezolid, pyrazinamide, and high-dose isoniazid. However, this is not without challenges and limitations – the role of isoniazid in MDR-TB is controversial, aminoglycosides are known to penetrate the BBB only if the meninges are inflamed, pyrazinamide needs to be administered in high doses to attain adequate concentrations in the CSF, and the CSF levels of fluoroquinolones are lower than those in serum despite excellent penetration^[^1^]^.

MRgFUS-mediated BBB disruption could facilitate enhanced intracranial penetration of poorly penetrating antituberculous drugs such as rifampicin, ethambutol, and streptomycin. However, no pharmacokinetic or efficacy data exist for MRgFUS-assisted delivery of anti-TB drugs, and this remains a hypothesis requiring validation.

## Role of MRgFUS

**Figure 3.**
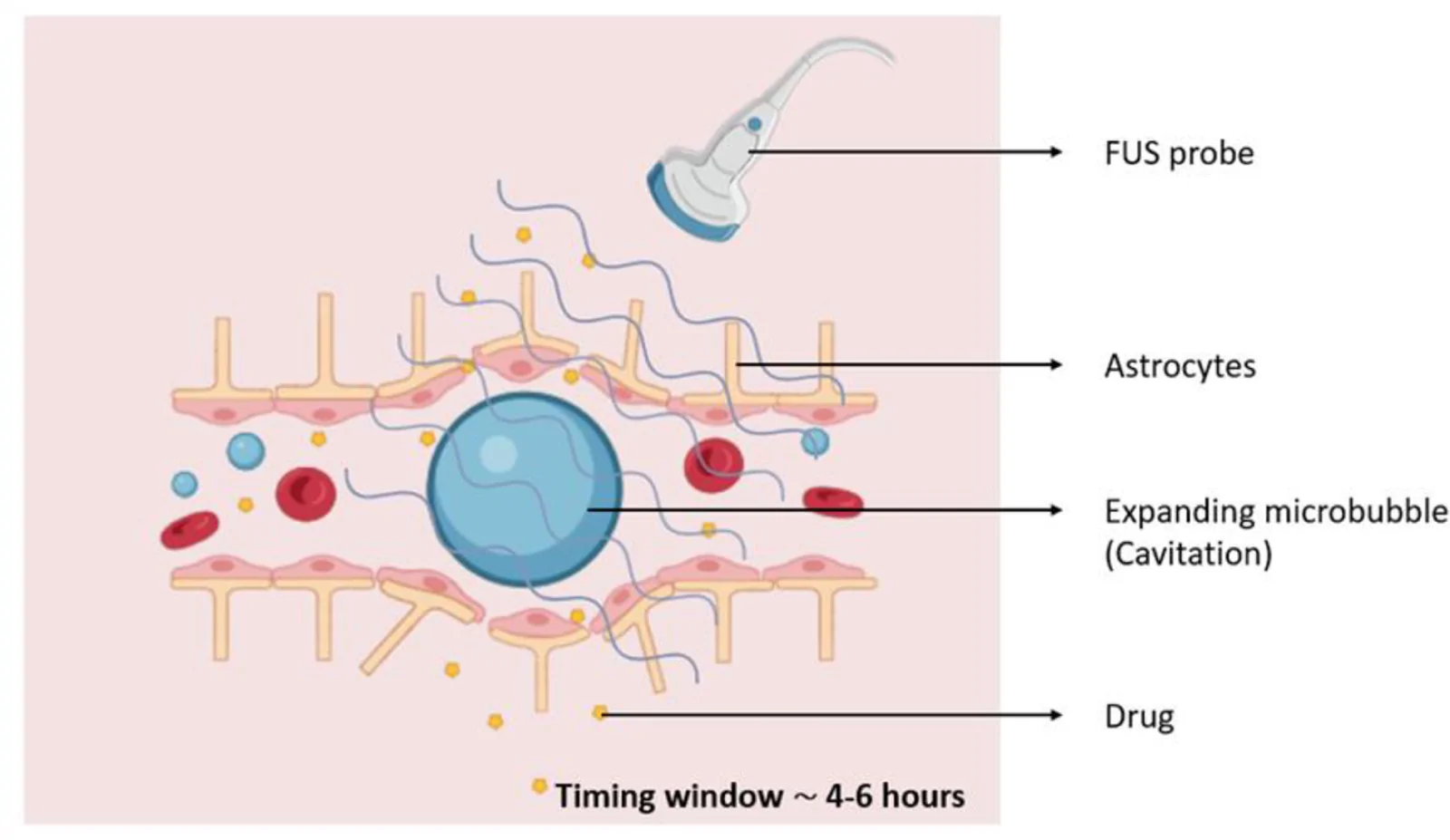
Focused ultrasound-induced disruption of the BBB and the BCSFB for enhanced drug delivery in CNS-TB.

Unless otherwise specified, MRgFUS in this review refers to the use of LIFU for transient BBB or BCSFB disruption rather than high-intensity thermal ablation. MRgFUS has been successfully used to achieve BBB opening with no tissue injury. The technique involves directing a focused ultrasonic beam that interacts with intravenously administered microbubbles^[^13^]^. These microbubbles are small spheres filled with high-molecular-weight gas and are injected peripherally into the bloodstream. When they pass through a region of sonication, they undergo conformational changes and oscillations, leading to the disruption of the BBB and increased vascular permeability^[^14^]^. This procedure is reversible, and the effects are transitory, lasting 4–6 hours, while also allowing for an effective yet more targeted delivery of the drug to the CNS^[^13,15^]^. Figure 3 illustrates a representative pulsed focused ultrasound protocol used to transiently disrupt the BBB.

## Preclinical and clinical data

Multiple animal studies have demonstrated that MRgFUS can significantly increase CNS drug levels, especially in the treatment of glioma^[^38^]^. Although most investigations have focused on oncology, emerging work suggests potential relevance for infectious diseases as well. Rieck *et al* reported that MRgFUS at 3 Hz significantly reduced bacterial counts in a mouse model of MRSA subcutaneous abscess^[^32^]^. In a rodent brain-abscess model, Liu *et al* showed that LIFU with microbubbles not only opens the BBB but also modulates the immune response, helping to contain the infection while reducing neuronal loss and vascular injury around the abscess^[^33^]^. Xie *et al* investigated the bactericidal effects of HIFU (1 MHz) on Bacillus Calmette-Guérin (BCG), both *in vitro* and *in vivo*, and concluded that it has therapeutic potential for treating BCG-infected tissues^[^31^]^.

## Drugs of interest

Rifampicin is one of the four main antitubercular drugs in the ATT regimen. However, it has very low penetration into the CSF, thereby requiring the administration of high doses to reach the minimum inhibitory concentration in the CSF^[^30^]^. Both ethambutol and streptomycin have poor penetration into the CSF^[^36^]^. A recent study has identified levofloxacin, ethionamide, pyrazinamide, and double doses of rifampicin and isoniazid as having better penetration into the CSF in patients with HIV ^[^39^]^. The newer drugs bedaquiline and delamanid, and the delamanid metabolite DM-6705, are highly bound to plasma proteins, which can limit their ability to cross the BBB and penetrate into the CSF^[^40–42^]^. The delivery of intrathecal anti-tubercular therapy can also be optimized using this technique (Fig. 3).

Focused ultrasound causes microbubble cavitation near endothelial walls, transiently increasing permeability and allowing drug entry into the brain parenchyma and CSF. The optimal window for drug delivery is approximately 4–6 hours. This was created by the authors based on data from literatures^[^13,14,43–45^]^.

A key advantage of MRgFUS is its precise targeting, facilitated by MR thermometry and contrast-enhanced imaging to monitor and guide sonication. This allows lesion-specific delivery, focusing on areas of disease activity while sparing healthy tissue^[^43^]^. Such localization mitigates the risk of widespread BBB disruption, which could otherwise predispose to neurotoxicity or edema. Importantly, the transient nature of barrier opening further enhances safety, with most animal models showing full BBB restoration within a few hours and no histological evidence of damage^[^44^]^. Table 1 summarizes the preclinical and clinical studies in which MRgFUS or HIFU was applied. Although CSF/plasma drug concentration ratios were not reported for anti-TB drugs, these studies demonstrate the potential of focused ultrasound to optimize therapy.

**Table 1:** Focused Ultrasound Applications in CNS and Infectious Disease Models Without Reported CSF/Plasma Drug Concentrations. Summary of preclinical studies using focused ultrasound (MRgFUS or HIFU) for infection control in CNS and non-CNS models. While no CSF/plasma drug ratios were reported, these studies highlight the adjunctive potential of focused ultrasound in reducing bacterial load, modulating immunity, and containing infection.

Drug (disease context)	Model (species)	Baseline CSF: Plasma	Post-FUS CSF: plasma	Source
Temozolomide (glioblastoma)	Rat, 9 L glioma	22.7%	38.6% (×1.7)	^[^46^]^
Carmustine (BCNU, glioma)	Human (IV labeling)	≥50%	≈100% (×2.0)	^[^47,48^]^
Bevacizumab (anti-VEGF, glioma)	Mouse, U87 glioma	~0.2%	~11% (×57)	^[^48,49^]^
Isoniazid (TB meningitis)	Human (TBM)	~0.86 (0.78–1.17 AUC ratio)	N/A	^[^50^]^
Pyrazinamide (TB meningitis)	Human (TBM)	~0.90 (AUC ratio)	N/A	^[^51^]^
Rifampicin (CNS TB, no inflammation)	Human (hydrocephalus)	~0.22 (0.13–0.42 AUC ratio)	N/A	^[^52^]^
Ethambutol (TB meningitis)	Human (TBM)	Very low	N/A	^[^49^]^
Linezolid (Gram⁺ CNS infection)	Human	~0.90 (0.80–1.0 AUC)	N/A	^[^50^]^

CSF ratios are expressed as % (CSF AUC ÷ plasma AUC) or as stated (AUC ratio).

## Inflammation in CNS-TB

IL-2 (a key T-cell growth factor) is produced by T cells (naive, Th1, and some CD8 +), and it promotes proliferation and differentiation of both T and B cells. IL-17A is produced by Th17 cells, CD8+ cells, natural killer (NK) cells, γδ T cells, and neutrophils. It stimulates endothelial cells and fibroblasts to release cytokines and antimicrobial peptides while also enhancing neutrophil production and recruitment. Together, IL-2 and IL-17A act as potent pro-inflammatory mediators.

IL-10, in contrast, is produced by macrophages, dendritic cells, and both B and T lymphocytes, and functions as a strong anti-inflammatory cytokine by suppressing macrophage activation and helping to regulate excessive immune responses^[^53,54^]^. Zaharie and colleagues found that patients who died from CNS-TB exhibited elevated levels of IL-2, IL-17A, and other inflammatory markers, indicating that excessive inflammation combined with insufficient immune regulation can lead to brain tissue damage, even after bacterial control has been achieved^[^55^]^.

A rare but possible complication of TB is vasculitis caused by infection with *Mycobacterium tuberculosis*, which can spread either by direct extension from pre-existing lesions (granulomas) or through hematogenous dissemination, resulting in vascular inflammation, luminal narrowing, thrombosis, and ischemic cerebral infarction^[^56,57^]^. The pathogenesis of TB-related vasculitis involves infiltrative, proliferative, and necrotizing processes in medium- and small-caliber intracranial vessels, leading to strokes and cerebral edema. Although corticosteroids and antiplatelet agents may be used for TB-associated cerebral vasculitis, no randomized clinical trials to date have demonstrated the efficacy or safety of these therapies in this context^[^57^]^.

## Role of MRgFUS in brain abscess

There is very limited evidence for the role of MRgFUS in inflammatory brain conditions such as brain abscesses. A study in an experimental brain abscess model showed that LIFU modulated the local immunological milieu and decreased the volume of the abscess, thereby suggesting a favorable adjuvant role for it in other brain lesions under controlled conditions^[^33^]^. However, no studies have evaluated this in human patients, and thus the possible complications of infection spread and increased edema cannot be ruled out.

## Immunomodulatory effects of MRgFUS in non-infectious CNS diseases

Evidence from oncology and neurodegenerative disease models suggests that MRgFUS can modulate immune cell recruitment and cytokine signaling^[^58–60^]^. Whether such immunomodulatory effects would be beneficial or harmful in CNS-TB is unknown. The concept of MRgFUS-facilitated delivery of anti-TNF agents or other immunotherapies in TBM remains speculative and should be considered hypothesis-generating only, given the lack of empirical data.

## MRgFUS-enabled delivery of immunotherapies: current evidence

Evidence supporting the use of MRgFUS for BBB opening – thereby enhancing the delivery of immunotherapeutics and other molecules – is promising. Preclinical studies have already demonstrated the superior efficacy of trastuzumab and pertuzumab in treating brain metastases from HER2-positive carcinomas in rats, the application of bevacizumab in a glioma xenograft model, and the use of bapineuzumab and solanezumab in rat models of AD via MRgFUS-mediated BBB opening^[^60,61^]^. The safety and feasibility of this approach have also been demonstrated in humans with advanced brain tumors^[^14^]^.

Building on these advances, early clinical studies have shown encouraging results, including improved outcomes with MRgFUS-enhanced trastuzumab delivery in four patients with HER2-positive breast cancer brain metastases and with MRgFUS-facilitated aducanumab delivery in three patients with Alzheimer’s disease^[^14,62,63^]^. Additionally, multiple ongoing trials are evaluating a broad range of immunotherapeutics, targeted agents, and stem cell-based therapies delivered through MRgFUS-induced BBB opening across diverse CNS disorders^[^14^]^.

In this way, MRgFUS has the potential not only to enhance immunotherapeutic delivery across the BBB but also to modulate and improve immune responses within the brain.

## Evidence gaps in CNS-TB and infectious conditions

Host-directed MRgFUS has been employed in cancer and neurological diseases, but its application in infectious diseases such as TB remains unexplored, representing a missed opportunity. To our knowledge, no studies to date have evaluated the effects of MRgFUS on outcomes in CNS-TB.

Additionally, no studies have investigated the use of MRgFUS to deliver anti-TNF agents. These drugs have the potential to mitigate TB-associated inflammation but have not yet been tested via this route, despite their promising therapeutic value. Anti-TNF agents can reduce deleterious inflammation in TBM, but they have limited penetration into the CNS. MRgFUS may facilitate targeted delivery of these agents, potentially reducing systemic side effects compared to corticosteroids, which, while effective in modulating inflammation in TBM, exert widespread effects throughout the body^[^64^]^. In contrast, MRgFUS could offer a more focused and safer therapeutic approach.

Furthermore, the technique may enhance the antibacterial activity of both the immune system and antibiotics through localized thermal effects, demonstrating potential in the management of infectious diseases^[^65^]^.

**Figure 4.**
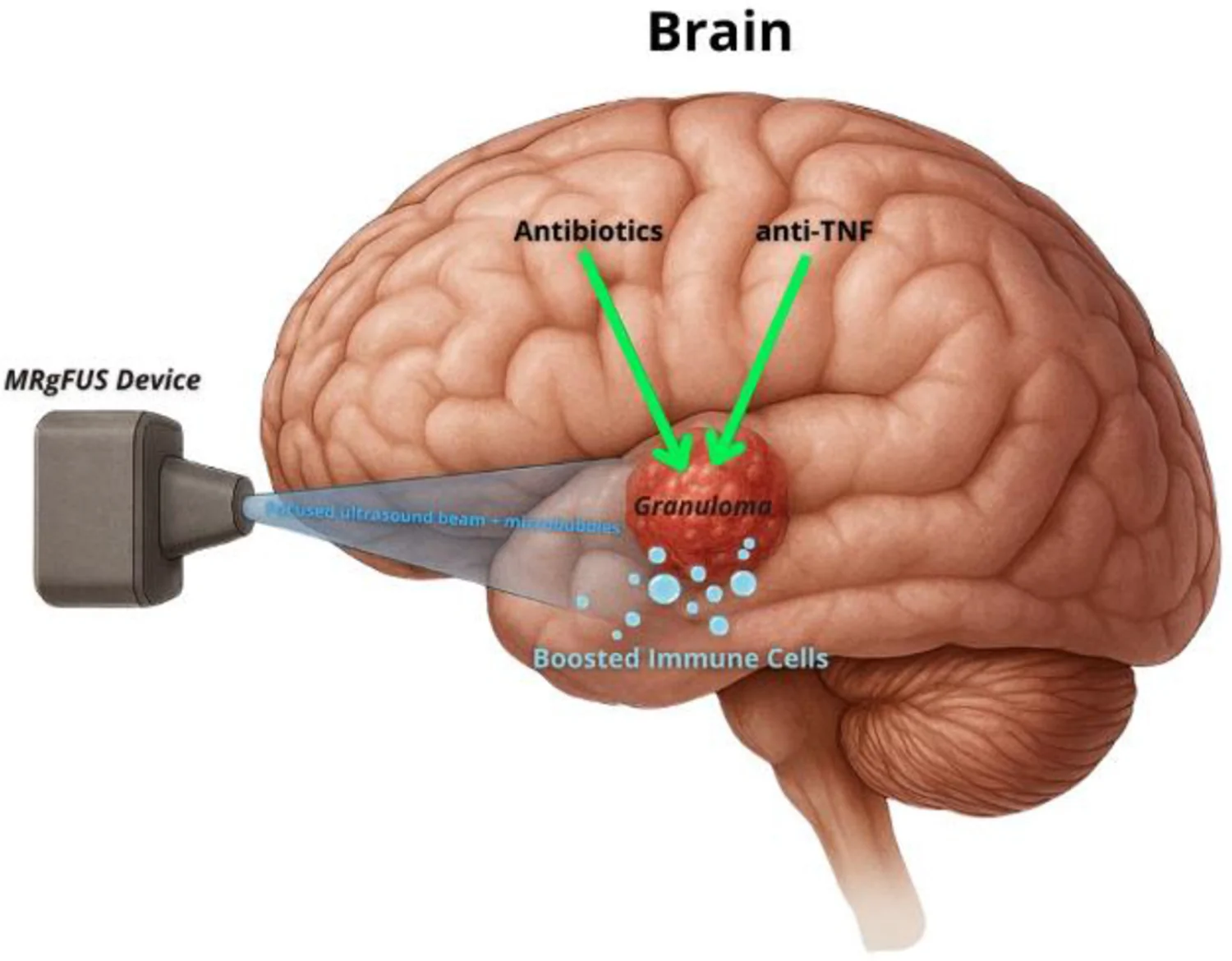
Postulated use of MRgFUS in TB of the brain.

Therefore, there is a significant opportunity for future studies to explore the therapeutic benefits of MRgFUS in CNS-TB and other neurological infectious diseases. This includes both the immunomodulatory and localized effects of the technique, as well as its capacity to enhance the delivery of antibiotics and immunotherapeutic agents across the BBB. For example, experimental research could assess the use of MRgFUS to facilitate anti-TNF therapy in animal models of TBM, helping to establish foundational evidence prior to clinical trials. MRgFUS thus represents a promising but not yet investigated strategy for improving immune modulation and drug delivery in CNS-TB. The proposed role of MRgFUS in CNS-TB is illustrated in Figure 4.

The figure illustrates the targeting strategy of MRgFUS for CNS tuberculoma. This approach involves the use of focused transcranial ultrasound with microbubbles to transiently disrupt the BBB, typically in combination with antibiotics and immunomodulators. MRgFUS may theoretically influence the fibrotic granuloma structure and locally modulate the immune microenvironment, although no experimental evidence currently supports direct granuloma disintegration in CNS-TB. This remains an area for conceptual and preclinical exploration.

While preclinical data on CNS-TB are lacking, the proposed ability of MRgFUS to “dismantle” or modulate the fibrotic granuloma matrix remains purely hypothetical. This concept is extrapolated from its proven thermomechanical effects in non-infectious, dense, and avascular tissues – such as uterine fibroids and intracranial tumors – where HIFU-induced coagulative necrosis and structural disruption have been documented^[^10–12^]^. Future studies are needed to determine whether similar effects could safely translate to CNS tuberculomas.

## Future directions, safety, and global relevance

The conceptualization of utilizing MRgFUS for the treatment of CNS-TB represents a paradigm shift in addressing an old yet persistently challenging infectious disease. For this hypothetical application to transition from bench to bedside, several critical steps in research, safety validation, and accessibility must be addressed.

## Future directions and research imperatives

Future research must prioritize preclinical validation in biologically relevant animal models of CNS-TB to establish clear therapeutic windows^[^66^]^. Key targets include optimizing sonication parameters to differentiate between non-thermal acoustic energy for BBB disruption and low-temperature thermal ablation to sterilize caseous necrosis within granulomas. Specific focus should be placed on investigating the synergy of MRgFUS-mediated drug delivery with current first-line anti-tubercular agents and novel host-directed therapies designed to modulate the immune microenvironment^[^67^]^. Rigorous investigation into the precise mechanisms by which MRgFUS may reprogram immune cell function, particularly within the perigranuloma tissue, is also required to maximize treatment efficacy and minimize relapse^[^68^]^. In addition, future pharmacokinetic and translational studies evaluating intracranial penetration of antitubercular agents following MR-guided focused ultrasound–mediated BBB modulation will be essential to determine the clinical feasibility, safety, and therapeutic benefit of this approach.

## Safety and regulatory considerations

Safety is paramount, given the invasive nature of CNS-TB lesions. The primary safety challenge lies in ensuring controlled, localized energy deposition in heterogeneous tissues, some of which may be calcified, necrotic, or highly inflamed^[^69^]^. Potential complications include uncontrolled thermal damage to surrounding healthy brain parenchyma, hemorrhagic transformation, and adverse immunological reactions following transient BBB disruption^[^70^]^. Clinical translation requires stringent safety protocols, including ultra-precise MR thermometry and sophisticated acoustic monitoring during every sonication^[^43^]^. Phase I human trials must initially focus on establishing the maximal safe energy delivery thresholds in patients with CNS-TB, ensuring that the benefits of a localized high drug concentration outweigh the procedural risks^[^71^]^.

## Global relevance

CNS-TB remains a leading cause of neurological morbidity and mortality, disproportionately affecting populations in LMICs^[^72^]^. Current treatment is often limited by poor drug penetration into granulomas and the CSF, contributing to treatment failure and drug resistance^[^73^]^. MRgFUS offers a technologically advanced solution to overcome these pharmacokinetic limitations. For the technology to achieve meaningful global impact, its integration into LMIC healthcare systems is essential (Table 2). This requires substantial effort to reduce the capital cost of MRgFUS hardware, simplify the clinical workflow, and establish sustainable partnerships to train local experts and ensure ongoing maintenance^[^74^]^. Only through this focused effort on cost-effectiveness and accessibility can MRgFUS realize its potential as a transformative tool in endemic regions. Table 2 summarizes key barriers to global MRgFUS deployment for CNS-TB and actionable opportunities to address them.

**Table 2:** Barriers and opportunities in deploying MRgFUS for CNS-TB Globally.

Theme	Barrier	Opportunity	Reference
Clinical	No completed clinical trials of MRgFUS in CNS-TB to date	Phase I/II trials Pharmocokinetics sampling, safety run-ins, and adaptive designs	^[^71,75^]^
	Risk of inflammatory flares or bacillary release with granuloma disruption	Protocolized antibiotic cover; staged sonications; real-time MRI/ultrasound feedback; predefined stopping rules	^[^69,76^]^
Technological	Limited MRI and MRgFUS availability in TB-endemic regions; scarce maintenance capacity	Lower-cost/portable FUS innovations; regional MRgFUS hubs with shared service contracts and vendor maintenance	^[^77,78^]^
	Unoptimized parameters for infected CNS lesions	TB-specific parameter-finding studies with standardized acoustic reporting and cavitation monitoring checklists	^[^79–81^]^
Economic	High capital and operating costs; limited payor coverage in LMICs	Pooled procurement; tiered pricing; cost-effectiveness studies leveraging avoided surgery/length of stay	^[^82^]^
Workforce	Shortage of trained radiologists/sonographers/neurosurgeons	Manufacturer- and society-led training; twinning programs; remote proctoring	^[^83,84^]^
Evidence & Adoption	Uncertain comparative effectiveness vs. standard care	Pragmatic trials vs. optimized medical/surgical care; core outcome sets meaningful to patients and health systems	^[^84^]^

## Limitations and knowledge gaps

Despite its theoretical appeal, the application of MRgFUS in CNS-TB faces substantial biological and translational uncertainties. A major concern is the potential risk of bacillary dissemination, meningeal seeding, or paradoxical inflammatory worsening following mechanical or thermal disruption of granulomatous lesions. Granulomas may function as partial containment structures; their disruption could theoretically facilitate the spread of *M. tuberculosis* into the surrounding brain parenchyma or CSF. Additionally, all mechanistic assumptions in this review are extrapolated from oncology, neurodegeneration, or brain abscess models. No *in vivo* CNS-TB models or human studies currently support the safety or efficacy of MRgFUS in this context. Differences in tissue composition, immune responses, and pathogen biology limit direct translation. Furthermore, transient BBB or BCSFB opening may provoke inflammatory cascades, edema, or hemorrhage, particularly in inflamed or vasculitic tissue. These risks underscore the necessity of stepwise preclinical validation in CNS-TB–specific animal models before any human application can be contemplated. Another important limitation is that the present work represents a narrative hypothesis-generating review rather than a systematic synthesis of the literature. Consequently, the selection of studies may be subject to narrative bias. Furthermore, the biological effects of focused ultrasound on mycobacterial granulomas and inflammatory immune responses remain poorly characterized, underscoring the need for dedicated preclinical and translational studies.

## Conclusion

CNS-TB remains one of the most devastating forms of tuberculosis, largely due to limited drug penetration across the BBB and the complex immunopathology of intracranial granulomas. MR-guided focused ultrasound represents a promising non-invasive technology capable of transiently modulating the BBB, enhancing targeted drug delivery, and potentially influencing granulomatous inflammatory processes. Although the applications proposed in this review remain hypothetical, they highlight an innovative interdisciplinary research direction integrating neurotherapeutics with infectious disease management. Future experimental and clinical studies are required to determine whether MR-guided focused ultrasound could emerge as a viable adjunctive strategy for improving treatment outcomes in CNS-TB.

Lastly, this narrative review adheres to the Transparency in the Reporting of Artificial Intelligence in Research (TITAN) guideline^[^85^]^. During the preparation of this manuscript, generative AI tools (DeepSeek, DeepSeek Inc., June 2025 version; temperature parameter = 0.7) were used only as auxiliary aids to assist in formatting, standardization, organization of background information, and language consistency checks. Furthermore, all AI-assisted text was thoroughly reviewed, edited, and refined by the authors. The conceptualization, literature interpretation, synthesis of ideas, and manuscript writing were performed entirely by the authors, ensuring academic rigor, accuracy, and originality of the review.

## Data Availability

No new datasets were generated or analyzed during the preparation of this review article. All information discussed in this manuscript is derived from previously published studies, which are appropriately cited in the reference list.

## Research registration unique identifying number (UIN)

Not applicable. This study is a narrative review and does not involve clinical trials, patient recruitment, or experimental interventions.

## References

[1] Katrak SM. Central nervous system tuberculosis. J Neurol Sci 2021;421:117278. 10.1016/j.jns.2020.117278 33387702

[2] Azeemuddin M Alvi A Sayani R. Neuroimaging findings in tuberculosis: a single-center experience in 559 cases. J Neuroimaging 2019;29:657–68. 10.1111/jon.12627 31115112

[3] Marx GE Chan ED. Tuberculous meningitis: diagnosis and treatment overview. Tuberc Res Treat 2011;2011:798764. 10.1155/2011/798764 PMC3335590 22567269

[4] Pandey PK Sharma AK Gupta U. Blood brain barrier: an overview on strategies in drug delivery, realistic in vitro modeling and in vivo live tracking. Tissue Barriers 2015;4:e1129476. 10.1080/21688370.2015.1129476 PMC4836458 27141418

[5] Perez-malagon CD Barrera-rodriguez R Lopez-gonzalez MA. Diagnostic and neurological overview of brain tuberculomas: a review of literature. Cureus 2021;13:e20133. 10.7759/cureus.20133 PMC8648135 34900500

[6] Ma Q Chen J Kong X. Interactions between CNS and immune cells in tuberculous meningitis. Front Immunol 2024;15:1326859. 10.3389/fimmu.2024.1326859 PMC10867975 38361935

[7] Lee EJ Fomenko A Lozano AM. Magnetic resonance-guided focused ultrasound: current status and future perspectives in thermal ablation and blood-brain barrier opening. J Korean Neurosurg Soc 2019;62:10–26. 10.3340/jkns.2018.0180 PMC6328789 30630292

[8] Gandhi K Barzegar-fallah A Banstola A. Ultrasound-mediated blood-brain barrier disruption for drug delivery: a systematic review of protocols, efficacy, and safety outcomes from preclinical and clinical studies. Pharmaceutics 2022;14:833. 10.3390/pharmaceutics14040833 PMC9029131 35456667

[9] Elias WJ Lipsman N Ondo WG. A Randomized Trial of Focused Ultrasound Thalamotomy for Essential Tremor. N Engl J Med 2016;375:730–39. 10.1056/NEJMoa1600159 27557301

[10] Siedek F Yeo SY Heijman E. Magnetic resonance-guided high-intensity focused ultrasound (MR-HIFU): technical background and overview of current clinical applications (Part 1). Magnetresonanz-gesteuerter Hochintensiver Fokussierter Ultraschall (MR-HIFU) 2019;191:522–30. 10.1055/a-0817-5645 30630200

[11] Kamimura HAS Sokolov A. Transcranial magnetic resonance-guided focused ultrasound for neurological applications: industry challenges, innovations, and future directions. J Neural Eng 2025;22:10.1088/1741–2552/adc8d2. 10.1088/1741-2552/adc8d2 40179892

[12] Coluccia D Fandino J Schwyzer L. First noninvasive thermal ablation of a brain tumor with MR-guided focused ultrasound. J Ther Ultrasound 2014;2:17. 10.1186/2050-5736-2-17 PMC4322509 25671132

[13] Alli S Figueiredo CA Golbourn B. Brainstem blood brain barrier disruption using focused ultrasound: a demonstration of feasibility and enhanced doxorubicin delivery. J Control Release 2018;281:29–41. 10.1016/j.jconrel.2018.05.005 PMC6026028 29753957

[14] Meng Y Kalia LV Kalia SK. Current progress in magnetic resonance-guided focused ultrasound to facilitate drug delivery across the blood-brain barrier. Pharmaceutics 2024;16:719. 10.3390/pharmaceutics16060719 PMC11206305 38931843

[15] Gasca-salas C Fernández-rodríguez B Pineda-pardo JA. Blood-brain barrier opening with focused ultrasound in Parkinson’s disease dementia. Nat Commun 2021;12:779. 10.1038/s41467-021-21022-9 PMC7859400 33536430

[16] Lipsman N Mainprize TG Schwartz ML. Intracranial applications of magnetic resonance-guided focused ultrasound. Neurotherapeutics 2014;11:593–605. 10.1007/s13311-014-0281-2 PMC4121456 24850310

[17] Rathi S Griffith JI Zhang W. The influence of the blood-brain barrier in the treatment of brain tumours. J Intern Med 2022;292:3–30. 10.1111/joim.13440 35040235

[18] Anderson NE Somaratne J Mason DF. Neurological and systemic complications of tuberculous meningitis and its treatment at Auckland City Hospital, New Zealand. J Clin Neurosci 2010;17:1114–18. 10.1016/j.jocn.2010.01.006 20605462

[19] Sy MCC Espiritu AI Pascual JLR 5th. Global frequency and clinical features of stroke in patients with tuberculous meningitis: a systematic review. JAMA Network Open 2022;5:e2229282. 10.1001/jamanetworkopen.2022.29282 PMC9437750 36048445

[20] Javaud N Certal RD Stirnemann J. Tuberculous cerebral vasculitis: retrospective study of 10 cases. Eur J Intern Med 2011;22:e99–e104. 10.1016/j.ejim.2011.04.004 22075322

[21] Tuberculous Meningitis International Research Consortium, Davis AG Nightingale S Springer PE. Neurocognitive and functional impairment in adult and paediatric tuberculous meningitis. Wellcome Open Res 2019;4:178. 10.12688/wellcomeopenres.15516.1 PMC6971841 31984243

[22] Gupta M Tobin EH Munakomi S. CNS Tuberculosis. In: StatPearls [Internet]. Treasure Island (FL): StatPearls Publishing; 2024. https://www.ncbi.nlm.nih.gov/books/NBK585138 36256788

[23] McMahon P Pisapia DJ Schweitzer AD. Central nervous system tuberculoma mimicking a brain tumor: a case report. Radiol Case Rep 2023;19:414–17. 10.1016/j.radcr.2023.10.042 PMC10679855 38028299

[24] Zumla A. Mandell, Douglas, and Bennett’s principles and practice of infectious diseases. Lancet Infect Dis 2010;10:303–04.

[25] Prideaux B Via LE Zimmerman MD. The association between sterilizing activity and drug distribution into tuberculosis lesions. Nat Med 2015;21:1223–27. 10.1038/nm.3937 PMC4598290 26343800

[26] Sarathy JP Zuccotto F Hsinpin H. Prediction of drug penetration in tuberculosis lesions. ACS Infect Dis 2016;2:552–63. 10.1021/acsinfecdis.6b00051 PMC5028112 27626295

[27] Chatterjee S. Brain tuberculomas, tubercular meningitis, and post-tubercular hydrocephalus in children. J Pediatr Neurosci 2011;6:S96–S100. 10.4103/1817-1745.85725 PMC3208909 22069437

[28] Monteith S Sheehan J Medel R. Potential intracranial applications of magnetic resonance-guided focused ultrasound surgery. J Neurosurg 2013;118:215–21. 10.3171/2012.10.JNS12449 23176339

[29] Bauer R Martin E Haegele-link S. Noninvasive functional neurosurgery using transcranial MR imaging-guided focused ultrasound. Parkinsonism Relat Disord 2014;20:S197–S199. 10.1016/S1353-8020(13)70046-4 24262180

[30] Mhambi S Fisher D Tchokonte MBT. Permeation challenges of drugs for treatment of neurological tuberculosis and HIV and the application of magneto-electric nanoparticle drug delivery systems. Pharmaceutics 2021;13:1479. 10.3390/pharmaceutics13091479 PMC8466684 34575555

[31] Xie S Cao H Li J. Bactericidal effects of high intensity focused ultrasound on bacillus calmette-guerin in vivo and in vitro. Int J Hyperthermia 2019;36:886–96. 10.1080/02656736.2019.1649474 31464154

[32] Rieck B Bates D Zhang K. Focused ultrasound treatment of abscesses induced by methicillin resistant staphylococcus aureus: feasibility study in a mouse model. Med Phys 2014;41:063301. 10.1118/1.4875692 24877838

[33] Liu ZH Chen NY Huang CY. Modulation of the immune response by focused ultrasound suppressed brain abscess formation. Drug Deliv Transl Res 2025. doi:10.1007/s13346-025-01847-3 40193008

[34] Pinzon RT Veronica V. Hydrocephalus caused by tuberculous meningitis in an immunocompetent young adult: a case report. Int Med Case Rep J 2023;16:187–92. 10.2147/IMCRJ.S389204 PMC10038010 36968269

[35] Alkins R Huang Y Pajek D. Cavitation-based third ventriculostomy using MRI-guided focused ultrasound. J Neurosurg 2013;119:1520–29. 10.3171/2013.8.JNS13969 PMC4080406 24074494

[36] Ko TH Lee YH Chan L. Magnetic resonance-guided focused ultrasound surgery for Parkinson’s disease: a mini-review and comparison between deep brain stimulation. Parkinsonism Relat Disord 2023;111:105431. 10.1016/j.parkreldis.2023.105431 37164870

[37] Madadi AK Sohn MJ. Comprehensive therapeutic approaches to tuberculous meningitis: pharmacokinetics, combined dosing, and advanced intrathecal therapies. Pharmaceutics 2024;16:540. 10.3390/pharmaceutics16040540 PMC11054600 38675201

[38] Bunevicius A McDannold NJ Golby AJ. Focused Ultrasound Strategies for Brain Tumor Therapy. Operative Neurosurg (Hagerstown, Md) 2020;19:9–18. 10.1093/ons/opz374 PMC7293897 31853548

[39] Alvarez-uria G Midde M Pakam R. Initial antituberculous regimen with better drug penetration into cerebrospinal fluid reduces mortality in HIV infected patients with tuberculous meningitis: data from an HIV observational cohort study. Tuberc Res Treat 2013;2013:242604. 10.1155/2013/242604 PMC3753756 23997952

[40] Mongia H Mamnoon F Silsarma A. Concomitant bedaquiline and delamanid therapy in patients with drug-resistant extra-pulmonary tuberculosis in Mumbai, India. J Clin Tuberc Other Mycobact Dis 2024;35:100433. 10.1016/j.jctube.2024.100433 PMC11015490 38617837

[41] Mazanhanga MT Joubert A Castel SA. Liquid chromatography-tandem mass spectrometry analysis of delamanid and its metabolite in human cerebrospinal fluid using protein precipitation and on-line solid-phase extraction. J Pharm Biomed Anal 2023;227:115281. 10.1016/j.jpba.2023.115281 PMC10023415 36739721

[42] Schaaf HS Seddon JA. Management of tuberculous meningitis in children. Paediatr Int Child Health 2021;41:231–36. 10.1080/20469047.2021.1952818 34783305

[43] Lipsman N Meng Y Bethune AJ. Blood-brain barrier opening in Alzheimer’s disease using MR-guided focused ultrasound. Nat Commun 2018;9:2336. 10.1038/s41467-018-04529-6 PMC6060168 30046032

[44] McDannold N Arvanitis CD Vykhodtseva N. Temporary disruption of the blood-brain barrier by use of ultrasound and microbubbles: safety and efficacy evaluation in rhesus macaques. Cancer Res 2012;72:3652–63. 10.1158/0008-5472.CAN-12-0128 PMC3533365 22552291

[45] Wang JB Di Ianni T Vyas DB. Focused ultrasound for noninvasive. Focal Pharmacol Neurointervention Front Neurosci 2020;14:675. 10.3389/fnins.2020.00675 PMC7372945 32760238

[46] Wei KC Chu PC Wang HY. Focused ultrasound-induced blood-brain barrier opening to enhance temozolomide delivery for glioblastoma treatment: a preclinical study. PloS One 2013;8:e58995. 10.1371/journal.pone.0058995 PMC3602591 23527068

[47] U.S. Food and Drug Administration. BICNU (carmustine) injection label [Internet]. Silver Spring (MD): FDA; 2011 [cited 2025 Sep 30]. https://www.accessdata.fda.gov/drugsatfda_docs/label/2011/017422s038lbl.pdf

[48] Johansen PM Hansen PY Mohamed AA. Focused ultrasound for treatment of peripheral brain tumors. Explor Drug Sci 2023;1:107–25.

[49] Pardridge WM. CSF, blood-brain barrier, and brain drug delivery. Expert Opin Drug Deliv 2016;13:963–75. 10.1517/17425247.2016.1171315 27020469

[50] Nau R Sörgel F Eiffert H. Penetration of drugs through the blood-cerebrospinal fluid/blood-brain barrier for treatment of central nervous system infections. Clin Microbiol Rev 2010;23:858–83. 10.1128/CMR.00007-10 PMC2952976 20930076

[51] Stemkens R Litjens CHC Dian S. Pharmacokinetics of pyrazinamide during the initial phase of tuberculous meningitis treatment. Int J Antimicrob Agents 2019;54:371–74. 10.1016/j.ijantimicag.2019.06.010 31202922

[52] Nau R Prange HW Menck S. Penetration of rifampicin into the cerebrospinal fluid of adults with uninflamed meninges. J Antimicrob Chemother 1992;29:719–24. 10.1093/jac/29.6.719 1506352

[53] Donald PR. Cerebrospinal fluid concentrations of antituberculosis agents in adults and children. Tuberculosis (Edinb) 2010;90:279–92. 10.1016/j.tube.2010.07.002 20709598

[54] Spanos JP Hsu NJ Jacobs M. Microglia are crucial regulators of neuro-immunity during central nervous system tuberculosis. Front Cell Neurosci 2015;9:182. 10.3389/fncel.2015.00182 PMC4435040 26041993

[55] Zaharie SD Franken DJ van der Kuip M. The immunological architecture of granulomatous inflammation in central nervous system tuberculosis. Tuberculosis (Edinb) 2020;125:102016. 10.1016/j.tube.2020.102016 33137697

[56] Valliappan S Indiran M Mallikarjuna SK. Atypical presentation of tuberculous vasculitis: dual large vessel occlusions leading to malignant ischaemic stroke. BMJ Case Rep 2024;17:e262681. 10.1136/bcr-2024-262681 39950653

[57] Carod Artal FJ. Clinical management of infectious cerebral vasculitides. Expert Rev Neurother 2016;16:205–21. 10.1586/14737175.2015.1134321 26689107

[58] Leporace M Calabria FF Siciliano R. The thermal ablation with MRgFUS: from physics to oncological applications. Cancers (Basel) 2024;17:36. 10.3390/cancers17010036 PMC11718996 39796667

[59] Zhang Y Wang J Ghobadi SN. Molecular identity changes of tumor-associated macrophages and microglia after magnetic resonance imaging-guided focused ultrasound-induced blood-brain barrier opening in a mouse glioblastoma model. Ultrasound Med Biol 2023;49:1082–90. 10.1016/j.ultrasmedbio.2022.12.006 PMC10059983 36717283

[60] Toccaceli G Delfini R Colonnese C. Emerging strategies and future perspective in neuro-oncology using transcranial focused ultrasonography technology. World Neurosurg 2018;117:84–91. 10.1016/j.wneu.2018.05.239 29890272

[61] Meng Y Suppiah S Mithani K. Current and emerging brain applications of MR-guided focused ultrasound. J Ther Ultrasound 2017;5:26. 10.1186/s40349-017-0105-z PMC5629772 29034095

[62] Meng Y Reilly RM Pezo RC. MR-guided focused ultrasound enhances delivery of trastuzumab to Her2-positive brain metastases. Sci Transl Med 2021;13:eabj4011. 10.1126/scitranslmed.abj4011 34644145

[63] Rezai AR D’haese PF Finomore V. Ultrasound blood-brain barrier opening and aducanumab in alzheimer’s disease. N Engl J Med 2024;390:55–62. 10.1056/NEJMoa2308719 38169490

[64] Lu HJ Guo D Wei QQ. Potential of neuroinflammation-modulating strategies in tuberculous meningitis: targeting microglia. Aging Dis 2024;15:1255–76. 10.14336/AD.2023.0311 PMC11081169 37196131

[65] Beserra A Pichardo S Kisselgoff D. Targeting feasibility evaluation of magnetic resonance-guided focused ultrasound in the management of osteomyelitis: a virtual treatment planning study in 75 patients. Int J Hyperthermia 2019;36:1012–23. 10.1080/02656736.2019.1663944 31544543

[66] Husain AA Gupta UD Gupta P. Modelling of cerebral tuberculosis in BALB/c mice using clinical strain from patients with CNS tuberculosis infection. Indian J Med Res 2017;145:833–39. 10.4103/ijmr.IJMR_1930_15 PMC5674554 29067986

[67] Ye D Luan J Pang H. Characterization of focused ultrasound-mediated brainstem delivery of intranasally administered agents. J Control Release 2020;328:276–85. 10.1016/j.jconrel.2020.08.053 PMC7749082 32871204

[68] McMahon D Hynynen K. Acute inflammatory response following increased blood-brain barrier permeability induced by focused ultrasound is dependent on microbubble dose. Theranostics 2017;7:3989–4000. 10.7150/thno.21630 PMC5667420 29109793

[69] Burgess MT Apostolakis I Konofagou EE. Power cavitation-guided blood-brain barrier opening with focused ultrasound and microbubbles. Phys Med Biol 2018;63:065009. 10.1088/1361-6560/aab05c PMC5881390 29457587

[70] Kovacs ZI Kim S Jikaria N. Disrupting the blood-brain barrier by focused ultrasound induces sterile inflammation. Proc Natl Acad Sci U S A 2017;114:E75–E84. 10.1073/pnas.1614777114 PMC5224365 27994152

[71] Park SH Kim MJ Jung HH. Safety and feasibility of multiple blood-brain barrier disruptions for the treatment of glioblastoma in patients undergoing standard adjuvant chemotherapy. J Neurosurg 2020;134:475–83. 10.3171/2019.10.JNS192206 31899873

[72] Thakur K Das M Dooley KE. The global neurological burden of tuberculosis. Semin Neurol 2018;38:226–37. 10.1055/s-0038-1651500 29791949

[73] Maranchick NF Alshaer MH Smith AGC. Cerebrospinal fluid concentrations of fluoroquinolones and carbapenems in tuberculosis meningitis. Front Pharmacol 2022;13:1048653. 10.3389/fphar.2022.1048653 PMC9791083 36578553

[74] Sclocco R Beissner F Bianciardi M. Challenges and opportunities for brainstem neuroimaging with ultrahigh field MRI. NeuroImage 2018;168:412–26. 10.1016/j.neuroimage.2017.02.052 PMC5777900 28232189

[75] Mainprize T Lipsman N Huang Y. Blood-brain barrier opening in primary brain tumors with non-invasive mr-guided focused ultrasound: a clinical safety and feasibility study. Sci Rep 2019;9. doi:10.1038/s41598-018-36340-0 PMC6344541 30674905

[76] Kaovasia TP Duclos S Gupta D. A pre-clinical MRI-guided all-in-one focused ultrasound system for murine brain studies. Sci Rep 2025;15:144. 10.1038/s41598-024-84078-9 PMC11696467 39747938

[77] Hu Z Chen S Yang Y. An affordable and easy-to-use focused ultrasound device for noninvasive and high precision drug delivery to the mouse brain. IEEE Trans Biomed Eng 2022;69:2723–32. 10.1109/TBME.2022.3150781 PMC9443669 35157574

[78] Hersh DS Kim AJ Winkles JA. Emerging applications of therapeutic ultrasound in neuro-oncology: moving beyond tumor ablation. Neurosurgery 2016;79:643–54. 10.1227/NEU.0000000000001399 PMC5073037 27552589

[79] O’reilly MA Hynynen K. Blood-brain barrier: real-time feedback-controlled focused ultrasound disruption by using an acoustic emissions-based controller. Radiology 2012;263:96–106. 10.1148/radiol.11111417 PMC3309801 22332065

[80] Kooiman K Roovers S Langeveld SAG. Ultrasound-responsive cavitation nuclei for therapy and drug delivery. Ultrasound Med Biol 2020;46:1296–325. 10.1016/j.ultrasmedbio.2020.01.002 PMC7189181 32165014

[81] Kohli M Korobitsyn A Ismail N. Correction: WHO target product profile for TB detection at peripheral settings: 2024 update. PLOS Global Public Health 2025;5:e0005315. 10.1371/journal.pgph.0005315 PMC12510484 41066338

[82] Meng Y Pople CB Kalia SK. Cost-effectiveness analysis of MR-guided focused ultrasound thalamotomy for tremor-dominant Parkinson’s disease. J Neurosurg 2020;135:273–78. 10.3171/2020.5.JNS20692 32764177

[83] Fowlkes B Ghanouni P Sanghvi N. International society for therapeutic ultrasound conference 2016: tel Aviv, Israel. 14-18 March, 2016. J Ther Ultrasound 2017;5:15.

[84] Millward CP Armstrong TS Barrington H. Opportunities and challenges for the development of “core outcome sets” in neuro-oncology. Neuro Oncol 2022;24:1048–55. 10.1093/neuonc/noac062 PMC9248398 35287168

[85] TITAN Group, Agha RA Mathew G Rashid R. Transparency in the reporting of Artificial Intelligence – the TITAN guideline. Prem J Sci 2025;10:100082.

